# Some Suggestions in Regard to Dental Examinations in the United States

**Published:** 1899-06

**Authors:** Waldo E. Royce

**Affiliations:** Tunbridge Wells, England


### SOME SUGGESTIONS IN REGARD TO DENTAL EXAMINATIONS IN THE UNITED STATES.[1]

BY WALDO E. ROYCE, D.D.S., TUNBRIDGE WELLS, ENGLAND.

A SUBJECT which has received so much of the best thought of our profession cannot be approached without a feeling of diffidence. Nothing short of the almost universal outcry against the system—or, rather, want of system—which now prevails in the United States in respect to dental education and examinations would tempt me to undertake so delicate a task. The thought has suggested itself, however, that, possibly, years of foreign residence may aid me in my effort.

Every thoughtful graduate of an American dental school looks with chagrin upon the lack of acknowledgment which is accorded his degree. He need not be an old man in order to remember the time when the degree of D.D.S. from a leading school was a professional passport in any part of the world. Now the doors of all Europe are shut against it; and, what is worse, it is not acknowledged in the very State where it was granted.

The American operator is able to give practical demonstration of the fact that, in manipulative ability, he still stands alone and supreme. With equal ease it can be proved that wherever conservative dentistry is practised nine-tenths of the methods and instruments used were conceived by Americans. American dental literature reaches all parts of Europe.

Notwithstanding this, all foreign states, and many of our own States, have made laws "to protect the public" from the ignorance of men who perform these operations, who conceive methods and appliances, or who edit journals and compile text-books that are gladly accepted the world over. All, so far as they are able to comprehend and use to their own advantage, accept the result of the

---

[1] Read before the American Dental Club, London, England.

thought, research, and ingenuity of the American dentist, and then, to show their gratitude, shut the door in his face.

Is, then, the creator so inferior to the works of his own creation? Have the teachers who are represented by the Association of Dental Faculties so betrayed their trust that they have brought disgrace upon themselves and upon the degree which they confer?

The men who edit our journals are nearly all teachers in our schools. Almost every prominent operator in the country is, more or less, directly connected with some school. Are all these men cheats and knaves? No fair-minded man believes this.

The trouble is that, unlike a gold filling, an ingenious instrument, or a printed journal, the work of the teacher cannot be seen, and at present there is no proper means of judging it.

Give our best dental schools a test by which they can be compared with each other, and with the schools of Europe, and they will soon restore their degree to the place which it once held and which it still deserves.

It must be admitted that all examinations are imperfect, but they are the best test we have of fitness, and, until we have something better, must be accepted. Being accepted, they should be made as perfect as possible. The student who has properly completed his curriculum has a right to an examination which shall be final and universally accepted. In all European countries this fact is recognized. The professional examinations are, more or less, directly under the supervision of the government, and the license which is issued bearing government sanction is of corresponding value. Of course, it is more difficult to arrange a government examination in a country made up of a large number of independent states, but this difficulty is not insurmountable.

When Uncle Sam wishes officers for his army and navy, he finds a way to look after their education. If it is legal for him to educate them, he surely can look to the examinations of dentists, lawyers, surgeons, engineers, etc., all of whom he has to employ.

This plan need not in any way interfere with State rights. If the several States are assured that the possessor of the national degree has passed an examination equal to, and probably more severe than, their own, they will readily amend their laws so as to exempt the holder of that degree from further examination.

It cannot be expected that all the States will at once bring their standard of requirements up to that of the government examination. Each one will have a right to fix any standard it may like, but it should be understood, both at home and abroad, that no degree, except that granted by the government, has any value outside the State where it was obtained. Soon the States would learn the value of the government examination, and would adopt its diploma as the only recognized qualification. A government examination would, I believe, also settle that vexed question, the undue increase of dental schools.

The Association of Dental Faculties has done a noble work in raising the standard of dental education in America, and it is worthy of all praise, but even in this Association the most progressive members complain that their advance is retarded by the less ambitious ones. Now, they can only say this *should* be required. Make them government examiners, and they will be able to say this *is* required.

Let any reasonable standard be established by a government board, and every school worthy of the name will be anxious to have its curriculum acknowledged. Each school will realize that to succeed it must be acknowledged, and that the only way to obtain students is to so educate them that they shall be able to pass the government examination. In fact, the examination would be of as much value in testing the schools as in testing the scholars. This must result in a healthy, friendly rivalry among the better class of schools and in the extermination of all others.

### A GOVERNMENT BOARD OF DENTAL EXAMINERS.

It is evident that the best interests of the dental profession, both at home and abroad, demand that the United States government should, through its proper agents, appoint a Board of Dental Examiners, and when such board is appointed the members should become United States officials, and should have power to grant diplomas in the name of the United States government.

A method of appointment should be conceived by which the best men in the profession shall be chosen as examiners, and the greatest care should be exercised that the office be placed beyond the reach of party or politicians.

The board should consist of at least five members (better ten), the first members to be appointed to act for one, two, three, four, and five years. At the end of each year a new member (or members) should be appointed to take the place of the retiring member (or members), to act for five years, thus making the work of the board practically continuous.

After five years no one should be eligible for appointment as a member of the examining board who has not passed the examination of the board.

To avoid confusion and an unnecessary increase of degrees, the successful candidate should be allowed to add the letter N (National) to his degree. The letters D.D.S.N. would show that a man had, in addition to taking his degree in a dental college, passed the government examination.

To falsely use the letters D.D.S.N. should be an indictable offence against the United States government, and should be punished with a heavy penalty.

The standard of requirements, both preliminary and professional, and the severity of the examination should at least equal those of the most exacting State in the Union. A schedule of these requirements should be published, together with a list of the dental schools whose curriculum is, for the time being, acknowledged by the board, and whose graduates are allowed to present themselves for examination.

This list of colleges should be revised by the board each year.

The board should require of each candidate for examination,—

*First.* A certificate of having passed the required preliminary examination. In case the candidate is not an American citizen, he should be required to produce a certificate of having passed the preliminary dental examination in his native country, in addition to such other certificates as the board may see fit to require.

*Second.* A diploma from a dental school which was at the time of granting the diploma recognized by the board.

*Third.* Each candidate should be required to sign an agreement that if he shall be granted the degree of the board, he will not advertise, or indulge in other unprofessional practice; that he received his degree upon this condition, and that he will forfeit his degree and surrender his diploma upon its being proved that he has broken this agreement.

Any person who holds the diploma of a reputable dental or medical school, granted before the formation of the Association of Dental Faculties, or who holds the diploma of a dental school, which diploma was granted before the formation of this board, and which school was at the time of granting said diploma a member of the Association of Dental Faculties, should, provided he can give proof of having been in *bona-fide* practice of dentistry within the limits of the United States for a period of not less than three years subsequent to receiving his diploma, be exempt from producing other certificates. The board should have no power to make further exemption than that above named, and the degree should in no case be granted as an honorary degree.

All examinations should be conducted in the English language and without interpreters.

In drawing this outline for the formation of a government examination, I do not suppose that it is perfect or complete. My object is to set the ball rolling in what I believe to be the right direction. This matter is too important and too difficult to be undertaken by one man. Time should be taken to enlist the hearty and active support of the best talent in the profession. With this help, a plan should be so perfectly conceived and so thoroughly worked out that when we approach the government and ask that an examining board be appointed, we shall know exactly what we wish; and when the board is formed, its instructions should be so explicit that everything, from the first, shall work smoothly and without the need of alteration.

At a meeting of "The American Dental Club of London, held April 3, 1899, it was, on motion, adopted,—

"That a hearty vote of thanks be accorded Dr. Royce for his paper proposing a federal diploma in the United States of America; also for the time and trouble he has expended in securing such eminent legal opinion as to the constitutionality of the proposition. And at the same time it was recommended that the paper, with counsel's opinion appended, be sent to the different dental journals for simultaneous publication."

The following legal opinions were given upon the foregoing paper:

OPINION OF MR. COGSWELL.

"I am asked whether an act of Congress establishing a board of examiners in dental science, with power to issue diplomas, would be constitutional.

"This question divides itself into two parts, or presents itself in two aspects:

"If by it is meant to inquire whether Congress would have the power to create such board and to require all persons desiring to follow the profession of dentistry in the United States to submit to such examination and to prohibit the practice of dentistry by persons not possessing such diplomas, I answer, No. Powers of Congress are conferred by Section 8 of Article I. of the Constitution, and the powers not delegated to Congress by the Constitution are reserved to the States or the people by the Tenth Amendment, adopted very shortly after the Constitution went into effect. It has been uniformly held that Congress can only exercise the powers thus designated by Section 8, or such as are reasonably necessary to carry them into effect. It is not necessary to enumerate those powers; it is sufficient to say that the establishment of a board of examiners in dental, medical, legal, or any other science, with the exclusive power to grant diplomas authorizing the holders thereof to practise their professions, is clearly not within the power thus conferred upon Congress. The practice of the several States is in conformity with this opinion, and the granting of diplomas in the various sciences, with the right to practise the corresponding professions, has been uniformly exercised by the respective States.

"If by this question it is meant to ask whether Congress has the power to create such a board as is described, with power to grant diplomas, leaving it entirely optional with any person to take such examination and not attempting to give to such examinations or diplomas any exclusive right, making them, so to speak, honorary, then I have no doubt that Congress has such power and may create such a board. I repeat that there may be no misapprehension, that such examinations would be purely voluntary and such diplomas purely honorary.

"It would of course be within the competency of the several States to make the obtaining of such diploma a condition precedent to the practice of dental science in such States, respectively, but they would then derive all their validity from the action of the State and not from that of Congress.

"WILLIAM F. COGSWELL."

OPINION OF MR. BURNS.

"I have carefully examined your paper entitled 'Some Suggestions in regard to Dental Examiners in the United States,' and the plan therein outlined for a government board of Dental Examiners. I think Congress has a right under the Constitution to establish a

Board of Dental Examiners and to authorize them to hold examinations and issue diplomas in the name of the United States government. Congress would not, however, have the power to make these examinations compulsory or to require the different States to recognize the diplomas granted by such board, but the power would remain in the different States to prescribe such examinations under State authority as might be deemed proper by the State Legislatures. Congress could provide that dentists must take the national examination to make them eligible for service in the army and navy or to entitle them to practise in the Territories of the United States.

"Respectfully yours,

"JOHN D. BURNS."

